# Autoimmunity associated with first psychotic episode. A Systematic review.

**DOI:** 10.1192/j.eurpsy.2023.2110

**Published:** 2023-07-19

**Authors:** E. De La Fuente Ruiz

**Affiliations:** Psiquiatría, Hospital Universitario Miguel Servet, Zaragoza, Spain

## Abstract

**Introduction:**

Autoimmunity mechanisms involve many cells that produce inflammatory cytokines which damage different organs, like the brain. There is a relationship between neuropsychiatric diseases, such as psychosis, and autoimmune diseases. In this article we try to demonstrate that treating autoimmune diseases appropriately improves clinical evolution of patients with a first psychotic episode.

**Objectives:**

The purpose of this article is to emphasize the importance of a multidisciplinary approach to a first psychotic episode. It is very important to perform autoimmunity tests to rule out secondary psychoses, even more so if the patient does not respond correctly to treatment with antipsychotics, to improve his/her prognosis and quality of life.

**Methods:**

We performed a literature search of PubMed database using the following MeSH terms: “Autoimmune Diseases” and “Psychotic Disorders”. 134 studies were published between 2017-2022. We selected the original papers that analyzed the association between autoimmune diseases and first psychotic episodes. Finally, 18 were selected.

**Results:**

In secondary psychoses, early diagnosis and treatment of the underlying pathology can lead to rapid improvement.

**Conclusions:**

A multidisciplinary approach is necessary from the first moment that a FPE is diagnosed, even more so in middle-aged women.

**Disclosure of Interest:**

None Declared

